# Dose- and Time-Dependent Effects of Cobalt Chloride Supplementation on Growth Performance and Intestinal Development in Weaned Piglets

**DOI:** 10.3390/ani16030440

**Published:** 2026-01-30

**Authors:** Min Wang, Siqi Li, Xin Wang, Yutong Zeng, Mingming Guo, Zhaobin Wang, Lanmei Yin, Qiye Wang, Jianzhong Li, Huansheng Yang

**Affiliations:** 1 Hunan International Joint Laboratory of Animal Intestinal Ecology and Health, Laboratory of Animal Nutrition and Human Health, College of Life Sciences, Hunan Normal University, Changsha 410081, China; wangmin202205@163.com (M.W.); lisiqi6682023@163.com (S.L.); 202210140259@hunnu.edu.cn (X.W.); hyfcxi@163.com (Y.Z.); 17707777138@163.com (M.G.); anywang0914@163.com (Z.W.); yinlanmei12@163.com (L.Y.); wangqiye@hunnu.edu.cn (Q.W.); ljzhong@hunnu.edu.cn (J.L.); 2 Hunan Provincial Key Laboratory of Animal Nutritional Physiology and Metabolic Process, Scientific Observing and Experimental Station of Animal Nutrition and Feed Science in South-Central, Ministry of Agriculture, Hunan Provincial Engineering Research Center for Healthy Livestock and Poultry Production, Key Laboratory of Agro-Ecological Processes in Subtropical Region, Institute of Subtropical Agriculture, Chinese Academy of Sciences, Changsha 410125, China

**Keywords:** cobalt chloride, growth performance, intestinal development, weaned piglet

## Abstract

The aim of our study was to evaluate the effect of dietary cobalt chloride (CoCl_2_) supplementation on diarrhea, growth performance, and intestinal development in post-weaning piglets. Twenty-six piglets weaned at 21 days of age (d 21) with similar body weights were randomly assigned to three treatments: a control group (*n* = 10), a low-dose CoCl_2_ group (1 mg/kg of diet; *n* = 8) and a high dose CoCl_2_ group (2 mg/kg of diet, *n* = 8). Piglets were housed individually and fed the experimental diets for 28 days, with a dietary transition at day 15. During the early post-weaning period (d0 to d14), dietary CoCl_2_ supplementation was associated with favorable trends in growth performance parameters, including ADG (average daily gain: linear, 0.05 < *p* < 0.1) and gain to feed ratio (linear, *p* < 0.05), as well as reduced fecal scores (Linear, *p* < 0.05). However, during the later post-weaning period (d15 to d28), increasing dietary CoCl_2_ levels were unfavorable trends in feed intake (Linear, *p* < 0.05) and ADG (Linear, 0.05 < *p* < 0.1). At the intestinal level, CoCl_2_ supplementation was associated with dose-related changes in intestinal morphology, epithelial cell differentiation, and luminal pH. Alterations were observed in duodenal crypt depth (CD) and ileal villus height (VH), and duodenal VH/CD (Linear, *p* < 0.05), without significant effects on ileal epithelial proliferation and apoptosis (*p* > 0.1). Changes in the numbers of goblet cells in villi (Quadratic, *p* < 0.05) and crypt (Linear, *p* < 0.05), and enteroendocrine cells (Quadratic, *p* < 0.05) in crypt exhibited dose-dependent trends. In addition, with the increase in the CoCl_2_ concentration, the expressions of genes related to nutrient transporters (*DMT1*, *GLUT2*, and *SGLT1*) and metabolism (*HIF*-*1α*, *FBP1*, and *FBP2*), as well as those related to the NOTCH signaling pathway (*LGR5*, *ATOH1*, *HES1*, and *NOTCH2*), showed a linear decrease (Liner, *p* < 0.05). This was the case except for *LDHA* and *DLL4* (Liner, *p* < 0.05). The expression of the former was the lowest in the high-dose group, while that of the latter was the lowest in the low-dose group. In vitro, CoCl_2_ exposure was associated with reduced organoid budding rates (Quadratic, *p* < 0.01), the budding numbers (Linear, *p* < 0.05) per organoid, and altered gene expression of *SGLT1* and *CHGA* (Linear, *p* < 0.05). In summary, dietary supplementation with CoCl_2_ exhibited dose- and time-dependent trends in weaned piglets. CoCl_2_ supplementation during the early post-weaning period (two weeks after weaning) was associated with favorable trends in growth performance and diarrhea, whereas prolonged supplementation (4 weeks after weaning) or higher dietary level (2 mg/kg of diet) were associated with unfavorable trends in growth performance and intestinal development. These findings suggest that CoCl_2_ may have potential as a short-term (two weeks after weaning), low-level (below 2 mg/kg diet) nutritional supplement, while caution is warranted regarding long-term supplementation or higher dietary inclusion levels.

## Simple Summary

Weaning is a critical period for piglets and is commonly associated with diarrhea, impaired intestinal function, and reduced growth performance. This study investigated the effects of dietary cobalt chloride supplementation on growth, diarrhea incidence, and intestinal development in weaned piglets. Piglets were fed diets containing no cobalt chloride, a low level, or a higher level for four weeks after weaning. During the first two weeks post-weaning, cobalt chloride supplementation was associated with favorable trends in growth performance and reduced diarrhea. In contrast, continuous supplementation for four weeks, particularly at the higher dietary level, was associated with reduced feed intake, slower growth, and unfavorable changes in intestinal development. These changes included alterations in intestinal morphology, epithelial cell differentiation, and the expression of genes involved in nutrient transport and energy metabolism. Overall, the results suggest that low-level cobalt chloride supplementation may be beneficial during the early post-weaning period, whereas prolonged supplementation or higher dietary inclusion levels should be applied with caution.

## 1. Introduction

In modern pig production, weaned piglets face numerous challenges. Weaning stress, including environmental changes and nutritional transitions, often leads to diarrhea, impaired intestinal function, and reduced growth performance, resulting in increased mortality and higher production costs [1,2,3]. During the weaning phase, piglets transition from sow’s milk to solid feed, placing considerable stress on the digestive and immune systems and frequently causing intestinal dysfunction that compromises nutrient digestion and absorption [3,4,5]. Trace elements such as zinc and copper have been reported to alleviate post-weaning diarrhea, potentially through modulation of gut microbiota and enhancement of antioxidant capacity [6,7,8]. However, the use of zinc oxide in pig feed was banned by the European Union in June 2022 due to concerns regarding environmental accumulation [9], prompting the search for alternative trace element additives.

Cobalt is an essential trace element and a core component of vitamin B12, playing a critical role in many metabolic processes [10,11]. Cobalt chloride (CoCl_2_), a commonly used cobalt source, has attracted attention for its multifaceted functions in animal nutrition, such as involvement in vitamin B_12_ synthesis and regulation of iron metabolism. Nevertheless, its nutritional effects remain controversial. While appropriate cobalt supplementation has been associated with improved growth performance in pigs [12], excessive intake has been linked to hepatorenal toxicity and metabolic disorders [10,13]. Although the maximum permitted dietary inclusion level of CoCl_2_ does not exceed 2 mg CoCl_2_/kg of diet in China (Announcement No. 2625, Ministry of Agriculture and Rural Affairs of the People’s Republic of China), limited information is available regarding the practical effects of sub-2 mg CoCl_2_/kg of diet in weaned piglets. In particular, the stage-specific and dose-dependent responses of growth performance, diarrhea incidence, and intestinal development to dietary CoCl_2_ supplementation remain poorly characterized. Moreover, the underlying regulatory mechanisms of intestinal development, including those involving the NOTCH signaling pathway, under CoCl_2_ exposure have not been fully elucidated. Therefore, this study aimed to evaluate the dose- and time-dependent effects of dietary CoCl_2_ supplementation on diarrhea, growth performance, and intestinal development in weaned piglets. A 28-day feeding trial integrating histological analyses, molecular assessments, and intestinal organoid models was conducted to provide a comprehensive evaluation of intestinal structural and functional responses. The findings are intended to provide a scientific basis for the rational and safe application of CoCl_2_ in pig production.

## 2. Materials and Methods

### 2.1. Animal Welfare Statement

The experimental design and procedures in our study were approved by the Animal Care Advisory Committee of Hunan Normal University (approval number: 2023–209), Changsha, Hunan Province. The care and use of animals followed the established standards outlined in the Guide for the Care and Use of Laboratory Animals [14].

### 2.2. Piglets Feeding Experiment

A total of 26 male piglets ([Yorkshire × Landrace] × Duroc) were weaned at 21 days with an initial body weight (BW) of 4.38 ± 0.54 kg and were randomly assigned to three treatments (one pig in a pen). Piglets were fed with a basal diet (0 mg CoCl_2_/kg of diet, 10 piglets) or a basal diet containing 1 mg CoCl_2_/kg of diet (8 piglets) or 2 mg CoCl_2_/kg of diet (8 piglets). The basal diet (Table 1) was formulated according to the nutrient requirements of swine [15]. The CoCl_2_ used in our study was supplied by Aladdin (Shanghai, China). CoCl_2_ was incorporated into the experimental diets using a stepwise premixing method to ensure homogenous distribution.

This trial lasted for 28 days. The BW of each pig was recorded on d0, d14, and d28. In addition, the daily feed intake per pen and diarrhea incidence were recorded every day. The average daily gain (ADG), average daily feed intake (ADFI), and G/F were calculated. The feces were scored on a 5-point scale: 1 = dry and hard stool, 2 = wet stool, 3 = mild diarrhea, 4 = severe diarrhea, and 5 = watery diarrhea [16].

### 2.3. Sample Collection

On day 29, all piglets (one piglet per pen) were sacrificed for sampling, with the order of sacrifice randomly determined within each treatment group after an overnight. Piglets were held under general anesthesia and euthanized by an intravenous (jugular vein) injection of 4% sodium pentobarbital solution (40 mg CoCl_2_/kg of diet BW). After the piglets were slaughtered, the intestines were immediately removed and divided into the duodenum, jejunum, ileum, caecum, and colon. Three centimeters of the anterior duodenum, the middle jejunum, and the posterior ileum were collected and placed in the formaldehyde fixation solution, and another 2 cm of the anterior duodenum, the middle jejunum, and the posterior ileum were collected to be put in liquid nitrogen and finally stored in a −80 °C freezer. The chyme from the stomach, jejunum, ileum, and colon was collected to measure the pH.

In addition, the stomach, liver, spleen, and kidneys were removed and weighed, the body height and body length were also measured, and the relative organ height/weight was calculated according to the ratio of organ to body length/weight.

### 2.4. Dietary Composition Analysis

In our study, according to the method of (AOAC, 2005) [17], we investigated the dry matter (DM), crude protein (CP), and gross energy (GE) in experimental diets. All analyses were performed on a DM basis. In brief, the DM concentration of the diet was measured by drying at 105 °C for 12 h, and the CP and GE were determined by an automatic nitrogen analyzer KDN-103 F (Shanghai Xianjian Instruments Co., Ltd., Shanghai, China) and a precision automatic calorimeter (Changsha Mingpeng Technology Co., Ltd., Changsha, China), respectively (Table 1).

### 2.5. Histological Assay

The fixed samples were embedded in paraffin wax and cut into 4 μm thick histological sections for hematoxylin and eosin staining (HE). The intestinal morphologies were collected under a microscope using a 10 x-combined magnification, and an image processing and analysis system (Version 1, Leica Imaging Systems Ltd., Cambridge, UK).

For immunohistochemistry, slides were dewaxed and rehydrated; endogenous peroxidases were inhibited with 3% hydrogen peroxide for 10 min at room temperature. Antigen retrieval was performed by boiling twice in a sodium citrate buffer (0.01 M, pH 6.0), three minutes each time. The nonspecific binding sites were blocked using a 1:9 dilution 5% bovine serum albumin (BSA; Boster Biological Technology Co., Ltd., Wuhan, China) incubation at 37 °C for 30 min. After samples were incubated with Ki67 antibody (Abcam, Shanghai, China. 1:800 dilution), cleaved caspase 3 (Cell Signaling Technology, 1:700 dilution), and Chromogranin A (ChgA) antibody (Abcam; 1: 600 dilution) at 37 °C for 90 min, sections were treated with goat anti-rabbit IgG secondary antibody (ZSGB-BIO, Beijing, China) for 50 min at 37 °C. Except for blocking the nonspecific sites, every step was followed by four 3 min washes in PBS (Phosphate-Buffered Saline). Then, the positive cells were visualized with a diaminobenzidine Kit (ZSGB-BIO, Beijing, China). A light microscope (Version 1, Leica Imaging Systems Ltd., Cambridge, UK) was used to capture 30 microscopic fields per sample under 20× magnification. Meanwhile, alcian blue-periodic acid Schiff reaction (AB-PAS) (Nanjing Jiancheng Bioengineering Institute, Nanjing, China) was performed to investigate the effect of CoCl_2_ on goblet cell population in the crypts and villi.

The positive cells were quantified using Image-Pro Plus 6.0 software (Media Cybernetics, Inc., Cambridge, MA, USA), following the method described in a previous study [18]. Scale bars were calibrated according to the microscope imaging system using the manufacturer-provided calibration parameters.

### 2.6. Detections of Enzyme Activities

The ileal tissue was homogenized in saline and centrifuged (2500× *g*, 4 °C, 10 min) to produce the supernatant. Lactase, maltase, glutathione peroxidase (GSH-PX), alkaline phosphatase (ALP), superoxide dismutase (SOD) (Nanjing Jiancheng Bioengineering Institute, Nanjing, China), sucrase, and malondialdehyde (MDA) (Suzhou Comin Biotechnology Co., Ltd., Suzhou, China) were determined using commercial kits following the manufacturers’ instructions. The total protein concentration of every sample was examined utilizing a protein assay kit (Abiowell Biotechnology Co., Ltd. Changsha, China).

### 2.7. RNA Extraction and Quantitative Real-Time PCR

Total RNA was extracted from frozen ileal tissue using RNAiso Plus (Takara, Dalian, China). The quality of RNA was checked by using 1% agarose gel electrophoresis. The concentration and purity of RNA were examined via a Synergy HTX Multi-Mode Reader (Biotech, Burlington, VT, USA). Then, a Prescript RT reagent kit with gDNA Eraser (Takara, Dalian, China) was used to synthesize cDNA according to the manufacturer’s instructions. Quantitative real-time PCR for gene expression was performed in duplicate using a TB Green quantitative PCR mix (Takara, Dalian, China) on the QuantStudio 5 Real-time PCR System analyzer (Thermo Fisher Scientific, Waltham, MA, USA). All primers were designed using National Center for Biotechnology Information [19] (Table 2). The fold change in the target genes was normalized to the housekeeping gene (β-actin) and calculated using the 2^−ΔΔCt^ method [20].

### 2.8. Organoid Treatment

The organoids were generated from the proximal jejunal crypts of 21-day-old piglets ([Yorkshire × Landrace] × Duroc). The crypt isolation and culture of piglets were performed according to [21]. Organoids were passaged every three days. To correspond to the dietary CoCl_2_ supplementation levels used in vivo (0, 1, and 2 mg of CoCl_2_/kg of diet), and based on a dose–conversion relationship between mg/kg (dietary concentration) and μg/mL (culture medium concentration), organoids were treated with 0, 1, or 2 μg/mL CoCl_2_ (Aladdin, Shanghai, China), respectively. In this in vitro experiment, we used a 24-well plate to explore the effect of CoCl_2_ on the organoids. Three replicates were performed per treatment, and each replicate contained approximately 400 organoids. Except for the content of CoCl_2_, the complete medium consisted of advanced DMEM/F12 (Gibco, New York, NY, USA), 1% Glutamax (Gibco), 1% HEPES (Gibco) and 1% penicillin/streptomycin (Gibco), Wnt3a, Noggin, and R-spondin1 conditioned medium, 1x N2 supplement (Gibco), 1x B27 (Gibco), n-Acetyl-L-Cysteine (Invitrogen, Carlsbad, CA, USA), 10 mM nicotinamide (Sigma-Aldrich, St. Louis, MO, USA), 50 ng/mL recombinant murine epidermal growth factor (Sigma-Aldrich), 0.5 μM A83-01 (Tocris, Bristol, UK), 3 μM SB202190 (R &D Systems, Minneapolis, MN, USA), 2.5 μM CHIR99021 (Sigma). The organoid images were taken at d2 and d3 under an inverted fluorescence microscope (Version 4.12, Leica Microsystem, Wetzlar, Germany). The organoid budding rate and the average budding numbers per organoid were calculated according to [21]. The organoid experiment was designed to assess short-term, dose-related responses to CoCl_2_ and was not intended to model long-term exposure effects.

### 2.9. Statistical Analysis

All data were analyzed using one-way analysis of variance (ANOVA) with dietary CoCl_2_ level as the fixed effect. Prior to analysis, the normality of residuals was assessed using the Shapiro–Wilk test, and data points exceeding ± standard deviations from the group mean were considered outliers and excluded. Homogeneity of variances was verified before further analysis. When a significant or marginal overall treatment effect was detected by ANOVA (*p* < 0.10), orthogonal polynomial contrasts were applied to evaluate linear and quadratic dose-related trends of dietary CoCl_2_ supplementation. These analyses were conducted to describe potential dose-dependent response patterns across the graded supplementation levels (0, 1, and 2 mg of CoCl_2_/kg of diet), as commonly adopted in nutritional studies. Given the limited number of dietary levels, the linear and quadratic contrasts were interpreted as trend analyses rather than formal regression modeling. Results are presented as means with pooled standard errors of the mean (SEM). Differences were considered statistically significant at *p* < 0.05, and tendencies were discussed when 0.05 ≤ *p* < 0.10.

## 3. Results

### 3.1. Biphasic Dose- and Time-Related Effects on Growth Performance and Diarrhea

From day 0 to day 14, G/F increased with increasing dietary CoCl_2_ levels, showing a significant linear trend (Table 3; Linear: *p* < 0.05). In addition, CoCl_2_ supplementation exhibited a tendency toward increased ADG during this period (Linear: 0.05 < *p* < 0.10), whereas ADFI was not affected (*p* > 0.05). During the first week post-weaning, fecal scores showed a quadratic tendency in response to CoCl_2_ supplementation (Table 3: 0.05 < *p* < 0.1). In contrast, during the second and third weeks, fecal scores decreased with increasing CoCl_2_ levels, exhibiting a significant linear trend (Linear: *p* < 0.05). However, from d 15 to d 28, increasing dietary CoCl_2_ levels were associated with a significant decreasing trend in ADFI (Linear: *p* < 0.05), and ADG also showed a decreasing tendency (Linear: 0.05 < *p* < 0.10). During the fourth week, no significant differences in fecal scores were observed among the treatment piglets group (*p* > 0.10).

In addition, increasing dietary CoCl_2_ levels were associated with a significant decreasing trend in absolute kidney weight (Table 4, *p* < 0.05). In addition, tendencies toward reduced body length, absolute weights, length of the large intestine, and spleen weight were observed with increasing CoCl_2_ supplementation (Linear: 0.05 < *p* < 0.10). No significant effect of CoCl_2_ supplementation was observed on small intestine length (*p* > 0.10). However, small intestine weight exhibited a (Quadratic: 0.05 < *p* < 0.10), with the highest value observed in the 1 mg of CoCl_2_/kg of diet. Relative organ indices were not affected by the dietary CoCl_2_ levels (Linear: *p* < 0.05).

### 3.2. Dose-Related Changes in Intestinal Morphology and Function

As the concentration of CoCl_2_ increased, the CD in the duodenum and the VH in the ileum showed a significant linear decrease, whereas the VH/CD in the duodenum increased linearly (Linear: *p* < 0.05). In addition, VH in the jejunum and the VH/CD in the ileum showed a linear decreasing trend (Linear: 0.05 ≤ *p* < 0.10; Table 5). The VW in both the duodenum and the jejunum showed significant quadratic responses to dietary CoCl_2_ levels (Quadratic: *p* < 0.05), with the lowest values observed in the high-dose group and the highest values in the low-dose group. Dietary CoCl_2_ supplementation did not affect the pH of gastric, jejunal and colonic contents (Table 4; *p* > 0.05); however, ileal pH exhibited a significant quadratic response (Quadratic; *p* < 0.05), with the lowest value observed in the 1 mg of CoCl_2_/kg of diet group, and the highest value in the 2 mg of CoCl_2_/kg of diet group. Digestive enzyme activities were generally unaffected by CoCl_2_ supplementation, except for maltase activity, which showed a decreasing tendency with increasing CoCl_2_ level (Linear: 0.05 < *p* < 0.1; Table 6). Antioxidant status indicators (SOD and MDA) exhibited significant quadratic responses (Quadratic: *p* < 0.05), with the highest values observed in the 1 mg of CoCl_2_/kg of diet group.

### 3.3. Changes in Gene Expression in Ileal Epithelial Tissue

Regarding the gene expressions related to nutrient transporter and metabolism, including glucose transporters (GLUT2 and SGLT1), divalent metal transporter 1 (DMT1), HIF-1α, and glucose metabolism (FBP1 and FBP2), all showed a linear decrease with increasing dietary CoCl_2_ level (Table 7; Linear: *p* < 0.05). However, the expression of the LDHA was highest in the 1 mg/kg group (Quadratic: *p* < 0.05). For the genes related to the Notch signaling pathway, the expressions of LGR5, ATOH1, HES1, and NOTCH2 genes all decreased with increasing doses of CoCl_2_ (Linear: *p* < 0.05). In addition, the addition of CoCl_2_ had a significant impact on the expression of DLL4 (Quadratic: *p* < 0.05) genes, with both genes exhibiting the lowest expression levels in the 1 mg/kg group. Nevertheless, the addition of CoCl_2_ did not have a significant effect on the expressions of NOTCH1 and DLL1 genes (*p* > 0.1).

### 3.4. Proliferation, Differentiation, and Apoptosis of Ileal Epithelial Cells

**Figure 1:**
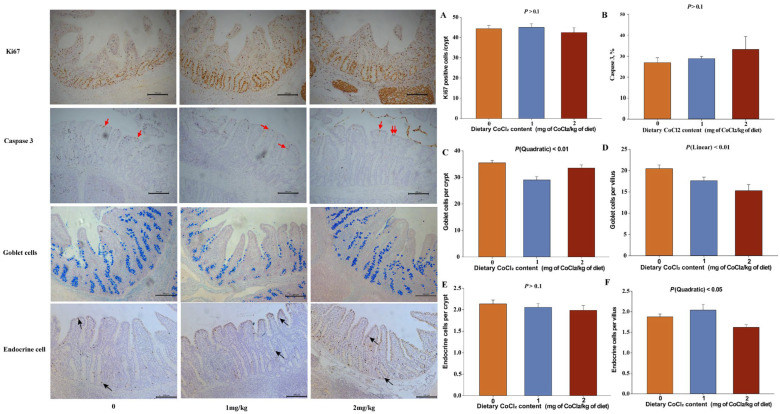
Effect of dietary CoCl_2_ supplementation of weaned piglets on ileal epithelial proliferation and differentiation at the end of the experimental period (28 d). (**A**) Ki67+ positive cells, a marker of cell proliferation; (**B**) cleaved caspase 3, a marker of cell apoptosis, as shown by the red arrow; (**C**,**D**) Goblet cells per crypt or villus; (**E**,**F**) enteroendocrine cells per crypt or villus, as shown by the black arrow. Differences among treatments were significant at *p* < 0.05; Scale bar = 200 μm (calibrated according to the microscope imaging system); Data are expressed as means ± SEM.

Immunohistochemical and AB-PAS staining results (Figure 1) indicated that four weeks of dietary CoCl_2_ supplementation did not significantly affect ileal epithelial tissue proliferation (Ki67, as shown in Figure 1A) and apoptosis (caspase3, as shown in Figure 1B) (*p* > 0.1). In contrast, epithelial cell differentiation exhibited dose-related responses. Goblet cells numbers in crypts showed a significant quadratic response to dietary CoCl_2_ supplementation (Figure 1C, Quadratic: *p* < 0.01), with the lowest values observed in the 1 mg of CoCl_2_/kg of diet group. In villi, goblet cell numbers showed a significant decreasing trend with increasing CoCl_2_ levels (Figure 1D; *p* < 0.05). Endocrine cell numbers in villi exhibited quadratic responses (Figure 1F, Quadratic: *p* < 0.5), with a significant increase observed in crypts (Figure 1E, *p* > 0.1).

### 3.5. Effect of CoCl_2_ on the Intestinal Organoid

**Figure 2:**
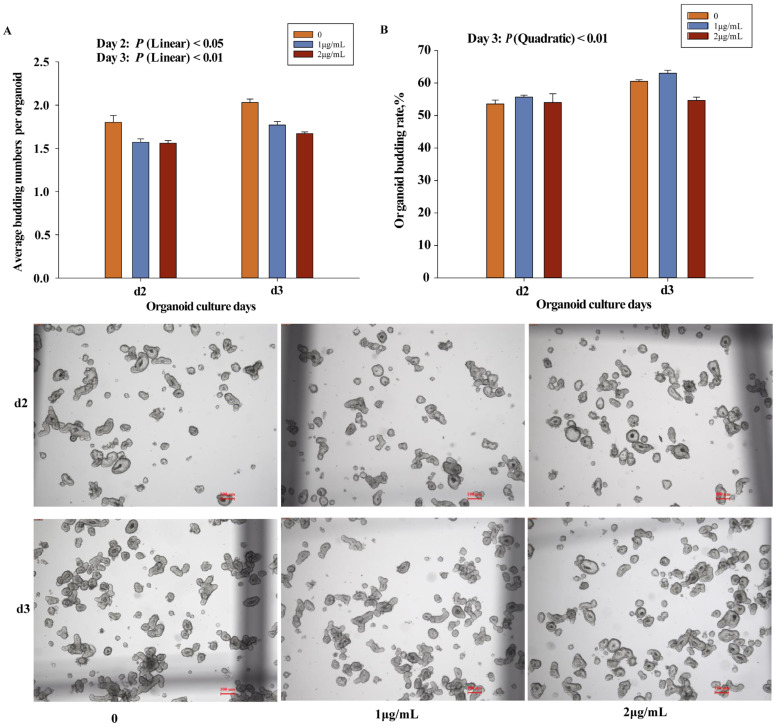
Effect of different concentrations of cobalt chloride on intestinal organoids of piglets. (**A**) Organoid budding rates. (**B**) Average number of buds per organoid. Organoids were treated with 0, 1, or 2 μg/mL CoCl_2_. Organoid images were acquired under a microscope at 20× combined magnification. Scale bars = 100 μm (*n* = 3 wells; calibrated according to the microscope imaging system). Data are expressed as means ± SEM; Differences among treatments were significant at *p* < 0.05.

In vitro experiments (Figure 2) showed that CoCl_2_ supplementation significantly reduced the number of buds per organoid, exhibiting quadratic responses (Figure 2A; Quadratic: *p* < 0.05), even at concentrations of 1 or 2 μg/mL. In addition, 2 μg/mL CoCl_2_ also reduced the organoid budding rate on day 3, showing a significant decreasing trend (Figure 2B, Linear: *p* < 0.01). Gene expression analysis in organoids (Table 8) showed that 2 μg/mL CoCl_2_ significantly reduced the expression of *SGLT* and *CHGA*, exhibiting decreasing trends with increasing CoCl_2_ concentrations (Linear: *p* < 0.05).

## 4. Discussion

This study aimed to explore the comprehensive effects of CoCl_2_ as a feed additive on the growth performance, diarrhea incidence, and intestinal development of weaned piglets. Weaning is a critical and challenging period in the life of piglets, often accompanied by digestive disorders, growth retardation, and diarrhea, which cause significant economic losses to the global pig farming industry [1]. Therefore, it is of great significance to find safe and effective feed additives to alleviate weaning stress and improve the health and production performance of piglets. Throughout this study, the effects of CoCl_2_ supplementation consistently exhibited dose- and time-related trends rather than definitive dose–response relationships. In the present in vivo feeding trial, “short-term” exposure refers specifically to the first 14 days post-weaning, whereas “long-term” exposure refers to continuous supplementation for 28 days. During the early post-weaning period (day 0–14), low-level dietary CoCl_2_ supplementation was associated with favorable trends in growth performance and diarrhea-related indicators, whereas prolonged exposure over 28 days, particularly at higher supplementation levels, was associated with unfavorable trends in feed intake, growth, and intestinal development.

### 4.1. Effects of Cobalt Chloride on the Growth Performance and Diarrhea of Weaned Piglets

In recent years, an increasing number of studies have indicated that inorganic elements can prevent and treat post-weaning diarrhea (PWD) in piglets by regulating intestinal function [22]. Previous research has demonstrated that zinc oxide can improve PWD through its antibacterial activity as well as by enhancing the function and structure of intestinal epithelial tight junctions, and copper sulfate can also serve as a growth-promoting feed additive, with its antibacterial activity reducing the bacterial population in the intestines of weaned piglets [23]. Cobalt is also an essential trace element, albeit less frequently present in metalloproteins compared to first-row metals such as iron, manganese, copper, or zinc [24,25]. Previous studies have suggested that CoCl_2_ can act as a hypoxia-mimetic agent to stabilize HIF-1α in rodent models [26,27]. Fisher, Khan [28] reported that maintenance of a hypoxic intestinal environment is closely associated with nutrient absorption, intestinal barrier function, and innate and adaptive immune responses in intestinal mucosal cells. Post-weaning diarrhea (PWD) typically occurs approximately one to two weeks after weaning in piglets [4,29], and is frequently associated with impaired intestinal barrier function [30]. We hypothesized that the damage to intestinal barrier function induced by weaning stress may be attributed to changes in the intestinal hypoxic environment. As expected, dietary CoCl_2_ supplementation was associated with improved growth performance and diarrhea-related indicators during the first two weeks post-weaning, a period when post-weaning diarrhea most frequently occurs. These effects were consistent with a dose- and time-dependent trend. Whether these effects are mediated through modulation of intestinal hypoxia remains to be clarified. In contrast, prolonged supplementation over four weeks was associated with unfavorable trends in feed intake, growth performance, and small intestine development, suggesting that excessive or extended exposure to CoCl_2_ may be accompanied by adverse physiological effects. These outcomes appeared to coincide with reduced feed intake, indicating that anorexia-related effects may contribute to the observed growth suppression, potentially in association with cobalt accumulation [13]. Supporting this interpretation, Skalny et al. reported that exposure to 75 mg CoCl_2_·6H_2_O per kg BW significantly reduced the weight of the kidneys in mice and was accompanied by altered tissue levels of Fe, Cu, Mn, and Zn, which were proposed as potential indicators of cobalt-related toxicity [31]. Although mineral concentrations were not directly measured in the present study, the observed reduction in kidney weight following four consecutive weeks of supplementation with 2 mg CoCl_2_/kg diet may reflect a similar pattern of indirect toxicological stress rather than a direct effect of CoCl_2_ on organ development. Taken together, the stage-specific responses observed in the present study suggest that the physiological effects associated with dietary CoCl_2_ supplementation are dependent on both dose and duration of exposure and are likely influenced by secondary changes in feed intake and nutritional status.

### 4.2. Effects of Cobalt Chloride on Intestinal Development and Its Molecular Mechanisms

This study found that dietary CoCl_2_ supplementation was associated with multifaceted alterations in intestinal development in weaned piglets. The small intestinal epithelium consists of millions of crypt-villus units, in which continuously dividing stem cells give rise to progenitor cells that ultimately differentiate into mature epithelial cells [32]. Maintenance of this dynamic structure is essential for normal intestinal function. Villous shortening has been reported to result from either increased epithelial cell loss or reduced epithelial renewal, often linked to decreased cell division or insufficient nutrient supply [33]. According to our data, alterations in small intestinal morphology were observed in association with dietary CoCl_2_ supplementation. These changes were closely related to feed intake during the later experimental period, suggesting that reduced nutrient intake may have contributed to villus atrophy and impaired epithelial maintenance. Similar associations between reduced energy intake and villus shortening have been reported previously [33]. Although Ki67 and caspase 3-positive cells were regarded as markers of cell proliferation and apoptosis, respectively [34], they were not significantly altered in the ileum; the reduced VH may reflect alternative mechanisms of epithelial loss, such as non-apoptotic cell extrusion. Indeed, previous studies have shown that intestinal epithelial homeostasis can be regulated through redundant pathways independent of caspase-3 and caspase-7 mediated apoptosis [35]. The absence of significant changes in digestive enzyme activities and antioxidant enzyme activities may also be related to the relatively short duration of dietary intervention.

Endocrine cells play a crucial role in the secretion of intestinal hormones, influencing digestion and metabolism [36]. Goblet cells are responsible for mucus secretion and are an essential component of the intestinal barrier function [37]. In this study, the numbers of these cell types exhibited dose-dependent associations with dietary CoCl_2_ supplementation, indicating a nonlinear pattern of epithelial differentiation. Moderate supplementation levels were associated with distinct cellular differentiation patterns, whereas higher supplementation levels were associated with less favorable cellular outcomes. These changes occurred concomitantly with reduced feed intake, suggesting that alterations in epithelial differentiation may be partially secondary to compromised nutritional status rather than solely attributable to direct effects of CoCl_2_. At the molecular level, piglets receiving 2 mg CoCl_2_/kg of diet exhibited lower expression of genes related to nutrient transport (*DMT1*, *GLUT2*, and *SGLT1*), metabolism, and glycolysis (such as *HIF-1α*, *LDHA*, *FBP1*, and *FBP2*). These reductions were closely associated with decreased feed intake at higher dietary CoCl_2_ levels, which likely limited energy availability in the intestine and contributed to the downregulation of metabolic pathways. While a direct regulatory effect of CoCl_2_ on glucose metabolism and hypoxia-related signaling cannot be completely excluded, the present findings suggest that the observed transcriptional changes are more likely indirect effects secondary to reduced nutrient intake. Previous studies have shown that HIF-1α plays a key role in regulating energy metabolism under hypoxic conditions, and excessive CoCl_2_ exposure may disrupt this balance rather than enhance adaptive responses [38]. However, from a toxicological perspective, these stage-dependent effects may also be influenced by cobalt accumulation and clearance dynamics in vivo. Although cobalt pharmacokinetics may lead to progressive tissue accumulation in the present study, prolonged dietary exposure may lead to progressive tissue accumulation, thereby indirectly affecting appetite, nutrient intake, and intestinal physiology. Further studies incorporating pharmacokinetic measurements are warranted to clarify these mechanisms.

Similarly, increasing dietary CoCl_2_ levels were associated with a progressive decline in the expression of genes related to the NOTCH signaling pathway. Research indicates that the NOTCH signaling pathway plays a pivotal role in the maintenance and differentiation of intestinal stem cells as well as the renewal of intestinal epithelial cells. Its inhibition may directly impact the integrity of intestinal structure and function [39]. *LGR5* is a marker of intestinal stem cells [40]. *ATOH1* is a key transcription factor for the differentiation of intestinal endocrine cells and goblet cells [41]; HES1 is a downstream target gene of the NOTCH signal, which typically inhibits the expression of *ATOH1* and promotes the differentiation of absorptive cells [42]. *NOTCH1* and *NOTCH2* are receptors of the NOTCH signaling pathway, while *DLL1* and *DLL4* are ligands of the NOTCH signaling pathway [32]. These gene changes may reflect impaired epithelial homeostasis during the later experimental period. Importantly, these transcriptional alterations coincided with reduced feed intake, suggesting that compromised nutritional status may have indirectly influenced NOTCH-related signaling and stem cell function. Therefore, the observed downregulation of NOTCH pathway components is more appropriately interpreted as an indirect consequence of excessive dietary CoCl_2_ exposure rather than a direct inhibitory effect. 

The intestinal organoid model is capable of mimicking the structure and function of the intestinal in vivo, and the decline in budding rate and number directly reflects the impaired proliferation of intestinal stem cells and the self-renewal capacity of epithelial cells [43,44]. In vitro organoid experiments further demonstrated that CoCl_2_ exposure was associated with reduced budding rate, the number of buds per organoid, and lower expression of *SGLT1* and *CHGA*. These findings indicate that CoCl_2_ can impair organoid growth and differentiation under controlled conditions. However, when interpreted together with the in vivo results, these effects should be considered supportive evidence rather than definitive proof of direct causality, particularly in light of the pronounced reduction in feed intake observed in vivo at higher dietary CoCl_2_ levels. Previous studies have shown that enteroendocrine cells may exhibit stem cell activity during homeostasis and injury-induced regeneration [45]. Therefore, the altered distribution of endocrine cells observed in this study may represent a compensatory response to impaired epithelial renewal, which appears to be insufficient under higher CoCl_2_ exposure.

## 5. Conclusions

In summary, this study demonstrates that dietary CoCl_2_ nutritional supplementation in weaned piglets exhibits dose-dependent responses and time-dependent effects in vivo. During the first two weeks post-weaning period, dietary CoCl_2_ supplementation was associated with favorable dose-related trends in growth performance and reduced diarrhea. In contrast, during the later post-weaning period (d 15 to d 28), increasing dietary CoCl_2_ supplementation levels were associated with unfavorable trends in growth performance, which may be related to impaired intestinal development, including alterations in the NOTCH signaling pathway and glycolytic metabolism.

In conclusion, these findings suggest that nutritional supplementation of CoCl_2_ may have potential as a short-term (first 14 days post-weaning), low-dose nutritional supplementation strategy, whereas caution is warranted regarding prolonged exposure (28 days) or higher supplementation levels. Future studies should further elucidate the dose- and duration-dependent effects of CoCl_2_ on intestinal development and clarify its regulatory role in intestinal stem cell activity and NOTCH signaling pathway, thereby providing a stronger scientific basis for the safe and effective application of CoCl_2_ in piglets’ nutrition.

## Figures and Tables

**Table 1:** The experiment of diet ingredients (as-fed basis).

Item	d 0 to 14	d 15 to 28
Ingredients, % as fed		
Corn grain grade 1	40.92	40.1
Extruded corn	20	22
Soybean protein concentrate	8	
Soybean meal, 43%NRC	8.2	20.5
Fish meal,63% CP	5	4
Whey powder	10	5
Soybean oil	0.5	1.5
Limestone	0.88	0.58
Dicalcium phosphate	0.5	0.88
Choline chloride	0.1	0.1
Antioxidants	0.05	0.05
Citric acid	0.8	0.5
Salt	0.1	0.1
Mineral mixture ^1^	0.15	0.15
Vitamin mixture ^2^	0.5	0.5
Lys 98%	0.64	0.53
DL-Met	0.36	0.27
L-Thr	0.24	0.19
L-Trp	0.06	0.05
Glucose	3	3
Total	100	100
Calculated nutrition level, %		
Net energy, Mcal/kg	2.49	2.45
CP, %	18.61	18.02
Calcium, %	0.8	0.7
Available Phosphorus, %	0.4	0.4
Lys ^3^	1.35	1.24
Met + Cys ^3^	0.77	0.68
Thr ^3^	0.79	0.73
Trp ^3^	0.22	0.22
Measured values, as DM basis ^4^		
DM, %	89.44	90.49
CP, %	20.86	19.49
GE, Mcal/kg	4.33	4.33

Note: ^1^ Provide the minimum of the following minerals per kilogram of diet: 100 mg ZnSO_4_, 30 mg MnSO_4_, 0.3 mg CoSO_4_, 150 mg FeSO_4_, 25 mg CuSO_4_, 0.3 mg Na_2_SeO_3_, and 0.5 mg KIO_3_. ^2^ Provide the minimum of the following vitamins per kilogram of diet: 2200 IU vitamin A, 17.5 mg vitamin B12, 220 IU vitamin D3, 16 IU vitamin E, 0.5 mg vitamin K3, 3.5 mg riboflavin, 30 mg niacin, 10 mg D-pantothenic acid, 0.05 mg biotin, 0.3 mg folic acid, 1.0 mg thiamine, 7 mg pyridoxine, and 4.0 mg ethoxyquin. ^3^ Standardized ileal digestible. ^4^ DM, dry matter; CP, crude protein; GE, gross energy.

**Table 2:** Primers for PCR.

Genes	Primers	Sequences (5′–3′)	Size, bp
*β-actin*	Forward	AGTTGAAGGTGGTCTCGTGG	215
	Reverse	TGCGGGACATCAAGGAGAAG	
*HIF-1A*	Forward	CTCCATTGCCTGCCTCTGAA	201
	Reverse	TGGGACTGTTAGGCTCAGGT	
*GLUT2*	Forward	AAGTCGAGGCCTATGATCTGACTAA	161
	Reverse	GGAAGAGGCATATCAGGACTCTACT	
*SGLT1*	Forward	ATCTCTGTCATCGTCATCTAC	121
	Reverse	GCCACCACACCATACTTC	
*LGR5*	Forward	GCCTTTGTAGGCAACCCTTC	121
	Reverse	AGGCACCATTCAAAGTCAGTG	
*ATOH1*	Forward	GGTGGTAGACGAGCTGGTTTG	170
	Reverse	CGTTGTTGAAGGACGGGATAA	
*HES1*	Forward	AAGCTGGAGAAGGCGGACAT	152
	Reverse	AAGCGGGTCACCTCGTTCAT	
*MUC2*	Forward	ACGCCATCCTGGGTGAGCT	121
	Reverse	ACGCTGCCGTCCGACTTGA	
*LYZ*	Forward	AATAGCCGCTACTGGTGTAATGATG	148
	Reverse	ATGCTTTAACGCCTAGTGGATCTCT	
*CHGA*	Forward	CCAGCACCCACCCCTTAGCC	192
	Reverse	CTTCTTCCTCCGGGACCGCC	
*LDHA*	Forward	GAAGTGCACTCCCGATTCCT	189
	Reverse	CGAGAGCAATTTCATCCGCC	
*FBP1*	Forward	CATCGCGCACCTCTATGGAA	127
	Reverse	GAGAACGCAGGTGGCAAAAG	
*FBP2*	Forward	CATGCTGACGGCCATCAAAG	72
	Reverse	GCGATTCCATACAGGTTGGC	
*DMT1*	Forward	CATCATCCCCACTCTGCTGG	109
	Reverse	GAGGACCAAGATACCGCCTG	
*NOTCH1*	Forward	ACAGCAACCCCTGTATCCAC	202
	Reverse	CAGTTGGGGCCGCTGAAG	
*NOTCH2*	Forward	AAACCTGGGAACAGAAGCACT	151
	Reverse	CTCGCAAGGGTCTCGATGT	
*DLL1*	Forward	CCTGACACTCAGGGGTGGAGA	248
	Reverse	TCAACACACAGGTGCCTCG	
*DLL4*	Forward	ATCCCCCACAATGGCTGTC	278
	Reverse	TAGCCATCCTCTTGGTCCTTGC	

Note: *DMT1*, solute carrier family 11 member 2; *GLUT2*, glucose transporter 2; *SGLT1*, sodium-glucose linked transporter 1; *HIF-1α*, hypoxia-inducible factor 1 subunit alpha; *LDHA*, lactate dehydrogenase A; *FBP1*, fructose-bisphosphatase 1; *FBP2*, fructose-bisphosphatase 2; *LGR5*, leucine-rich repeat-containing G protein-coupled receptor; *CHGA*, chromogranin A; *LYZ*, lysozyme; *ATOH1*, atonal bHLH transcription factor 1; Hes1, hairy enhancer of split 1; *MUC2*, mucin 2; *NOTCH1*, notch receptor 1; *NOTCH2*, notch receptor 2; *DLL1*, delta-like canonical Notch ligand 1; *DLL4*, delta-like canonical Notch ligand 4.

**Table 3:** Effect of dietary CoCl_2_ supplementation on growth performance and fecal score of weaned piglets.

Item	mg of CoCl_2_/kg of Diet			SEM	*p*-Value	
	0	1	2		Linear	Quadratic
1d BW, kg	4.5	4.3	4.4	0.12	0.743	0.624
14 d BW, g	5.30	5.26	5.66	0.167	0.401	0.555
28 d BW, kg	9.60	9.51	9.26	0.247	0.589	0.878
d0 to d14						
ADG, g	58.21	64.76	85.71	6.103	0.067	0.572
ADFI, g	253.02	260.03	248.56	6.146	0.776	0.508
G/F	0.23	0.25	0.33	0.020	0.032	0.363
d15 to d28						
ADG, g	301.07	283.81	240.00	13.004	0.056	0.622
ADFI, g	446.60	415.35	367.60	14.106	0.02	0.767
G/F	0.67	0.69	0.66	0.021	0.831	0.595
Fecal score						
First week	1.94	1.23	1.70	0.060	0.481	0.06
Second week	1.62	1.59	0.96	0.100	0.013	0.206
Third week	1.42	0.95	0.49	0.258	0.022	0.991
Fourth week	0.20	0.50	0.25	0.200	0.892	0.402

Note: BW, body weight; ADFI, average of daily feed intake; ADG, average daily gain; G/F, ADG/ADFI. Differences among treatments were significant at *p* < 0.05.

**Table 4:** Effect of dietary CoCl_2_ supplementation of weaned piglets on organ index and pH of intestinal contents at the end of the experimental period (28 d).

Item	mg of CoCl_2_/kg of Diet			SEM	*p-*Value	
	0	1	2		Linear	Quadratic
Body height, cm	34.56	34	33.25	0.515	0.317	0.932
Body length, cm	49	46.13	45.5	0.772	0.061	0.488
Absolute Values						
Small intestine length, cm	1238.1	1191.75	1152.25	29.174	0.241	0.958
Small intestine weight, g	500.83	538.88	449.38	15.734	0.159	0.053
Large intestine length, cm	286.4	284.75	259.75	5.747	0.055	0.334
Large intestine weight, g	233.86	225.66	205.74	6.366	0.072	0.665
Liver weight, g	299.37	311.43	262.45	9.902	0.119	0.15
Spleen weight, g	23.99	20.27	20.06	0.95	0.086	0.391
kidney weight, g	61.24	57.54	51.7	1.719	0.024	0.759
Stomach weight, g	86.8	90.44	81.7	2.75	0.455	0.315
Relative Value						
Small intestine, cm/cm body length	25.39	25.85	25.29	0.559	0.942	0.688
Small intestine, g/kg BW	52.9	54.83	51.57	1.237	0.666	0.352
Large intestine, cm/cm body length	5.87	6.21	5.73	0.137	0.666	0.175
Large intestine, g/kg BW	23.95	22.93	24.05	0.658	0.951	0.474
Liver, g/kg BW	30.47	31.6	30.17	0.631	0.852	0.37
Spleen, g/kg BW	2.44	2.09	2.57	0.113	0.625	0.103
kidney, g/kg BW	6.06	5.89	5.39	0.277	0.343	0.797
Stomach, g/kg BW	8.91	9.14	9.34	0.197	0.38	0.977
pH of Intestinal Chyme						
Stomach	2.2	2.65	2.23	0.187	0.941	0.302
Jejunum	6.33	6.67	6.44	0.103	0.659	0.205
Ileum	7.09	6.68	7.29	0.092	0.328	0.008
Colon	6.34	6.36	6.27	0.04	0.478	0.505

Note: Differences among treatments were significant at *p* < 0.05.

**Table 5:** Effect of dietary CoCl_2_ supplementation of weaned piglets on intestinal morphology at the end of the experimental period (28 d).

Item	mg of CoCl_2_/kg of Diet			SEM	*p-*Value	
	0	1	2		Linear	Quadratic
Duodenum						
VH, μm	335.32	341.71	303.85	8.702	0.145	0.23
CD, μm	410.89	363.44	302.05	14.264	0.001	0.777
VW, μm	136.97	146.46	132.61	2.681	0.491	0.041
VH/CD	0.83	0.96	1.01	0.03	0.012	0.511
Jejunum						
VH, μm	363.12	331.46	321.51	10.096	0.088	0.619
CD, μm	257.03	269.38	262.54	6.343	0.727	0.513
VW, μm	124.03	134.15	123.88	2.312	0.978	0.047
VH/CD	1.42	1.24	1.25	0.043	0.106	0.318
Ileum						
VH, μm	366.47	338.51	304.02	9.484	0.006	0.851
CD, μm	248.7	262.33	239.47	6.876	0.595	0.222
VW, μm	125.54	127.37	126.83	2.704	0.857	0.844
VH/CD	1.48	1.33	1.27	0.046	0.076	0.643

Note: VH, villous height; VW, villous width; CD, crypt depth. Differences among treatments were significant at *p* < 0.05.

**Table 6:** Effect of dietary CoCl_2_ supplementation of weaned piglets on ileal digestive enzymes and antioxidant enzymes at the end of the experimental period (28 d).

Item	mg of CoCl_2_/kg of Diet			SEM	*p-*Value	
	0	1	2		Linear	Quadratic
Digestive enzymes						
Maltase, U/mgprot	24.96	28.5	20.11	1.875	0.285	0.142
Lactase, U/mgprot	2.77	3.08	1.75	0.256	0.094	0.128
ALP, king unit/gprot	45.7	55.46	64.13	5.275	0.164	0.962
Scrase, ug/min/mgprot	25.51	25.74	27.13	1.052	0.549	0.805
Antioxidant status						
SOD, U/mgprot	1092.12	1172.82	1101.56	18.288	0.827	0.055
GSH-PX, enzyme active unit	148.25	142.03	133.89	4.974	0.253	0.93
MDA, nmol/mg prot	0.17	0.2	0.14	0.011	0.259	0.058

Note: ALP, alkaline phosphatase; SOD, superoxide dismutase; GSH-PX, glutathione peroxidase; MDA, malondialdehyde. Differences among treatments were significant at *p* < 0.05.

**Table 7:** Effect of dietary CoCl_2_ supplementation of weaned piglets on relative expression of ileal genes at the end of the experimental period (28 d).

Item	mg of CoCl_2_/kg of Diet			SEM	*p-*Value	
	0	1	2		Linear	Quadratic
Nutrient transporters and metabolism-related genes						
*DMT1*	0.98	0.86	0.77	0.03	0.01	0.75
*GLUT2*	1.03	0.68	0.53	0.073	0.003	0.461
*SGLT1*	1.07	0.85	0.71	0.078	0.055	0.785
*HIF-1α*	1.01	0.72	0.71	0.04	<0.01	0.04
*LDHA*	1.11	1.37	0.74	0.074	0.011	0.001
*FBP1*	0.97	0.83	0.57	0.049	<0.001	0.446
*FBP2*	1.02	0.75	0.58	0.05	<0.001	0.56
Notch signaling pathway components						
*LGR5*	1.05	0.67	0.7	0.067	0.027	0.124
*ATOH1*	1.02	0.77	0.71	0.046	0.002	0.241
*HES1*	1.01	0.88	0.76	0.035	0.001	0.92
*DLL1*	1.02	0.97	0.88	0.049	0.257	0.868
*DLL4*	0.85	0.6	1.18	0.073	0.031	0.003
*NOTCH1*	0.96	1.33	1.17	0.08	0.118	0.118
*NOTCH2*	0.97	0.81	0.8	0.034	0.039	0.308

Note: *DMT1*, solute carrier family 11 member 2; *GLUT2*, glucose transporter 2; *SGLT1*, sodium-glucose linked transporter 1; *HIF*-*1α*, hypoxia-inducible factor 1 subunit alpha; *LDHA*, lactate dehydrogenase A; *FBP1*, fructose-bisphosphatase 1; *FBP2*, fructose-bisphosphatase 2; *LGR5*, leucine-rich repeat-containing G protein-coupled receptor; *ATOH1*, atonal bHLH transcription factor 1; *HES1*, hairy enhancer of split 1; *NOTCH1*, notch receptor 1; *NOTCH2*, notch receptor 2; *DLL1*, delta-like canonical notch ligand 1; *DLL4*, delta-like canonical notch ligand 4. Differences among treatments were significant at *p* < 0.05.

**Table 8:** Effect of cobalt chloride concentration on intestinal stem cell activity in piglets.

Item	Supplement CoCl_2_ Level, μg/mL			SEM	*p-*Value	
	Con	1	2		Linear	Quadratic
*GLUT2*	2.55	1.32	1.04	0.714	0.713	0.782
*SGLT1*	1.01	0.67	0.75	0.065	0.044	0.068
*HIF-1α*	1.01	0.74	0.75	0.066	0.16	0.282
*LDHA*	1.04	0.68	0.69	0.085	0.128	0.249
*LGR5*	1.18	0.46	0.93	0.185	0.311	0.163
*LYZ*	1.04	1.18	1.26	0.082	0.624	0.865
*MUC2*	1	1.03	0.97	0.029	0.719	0.502
*CHGA*	1	0.96	0.84	0.031	0.027	0.444

Note: *GLUT2*, glucose transporter 2; *SGLT1*, sodium-glucose linked transporter 1; *HIF-1α*, hypoxia-inducible factor 1 subunit alpha; *LDHA*, lactate dehydrogenase A; *LGR5*, leucine-rich repeat-containing G protein-coupled receptor; *MUC2*, mucin 2; *LYZ*, lysozyme; *CHGA*, chromogranin A. Differences among treatments were significant at *p* < 0.05.

## Data Availability

All data are contained in the manuscript. Please contact the corresponding author for additional data requests.
